# Operator gender differences in major mechanical complications after central line insertions: a subgroup analysis of a prospective multicentre cohort study

**DOI:** 10.1186/s12871-024-02455-3

**Published:** 2024-02-21

**Authors:** Leila Naddi, Janna Hübinette, Thomas Kander, Ola Borgquist, Maria Adrian

**Affiliations:** 1 Anaesthesiology and Intensive Care, Department of Clinical Sciences, Lund University, Lund, Sweden; 2 Department of Intensive and Perioperative Care, Skåne University Hospital, 221 85 Lund, Sweden; 3 Medical Faculty, Lund University, Lund, Sweden; 4 Department of Cardiothoracic Surgery, Anaesthesia and Intensive Care, Skåne University Hospital, Lund, Sweden

**Keywords:** Central venous catheters, Catheterisation, central venous, Mechanical complication, Male, Female

## Abstract

**Background:**

A previous study on mechanical complications after central venous catheterisation demonstrated differences in complication rates between male and female operators. The objective of this subgroup analysis was to further investigate these differences. The hypothesis was that differences in distribution of predefined variables between operator genders could be identified.

**Methods:**

This was a subgroup analysis of a prospective, multicentre, observational cohort study conducted between March 2019 and December 2020 including 8 586 patients ≥ 16 years receiving central venous catheters at four emergency care hospitals. The main outcome measure was major mechanical complications defined as major bleeding, severe cardiac arrhythmia, pneumothorax, arterial catheterisation, and persistent nerve injury. Independent t-test and χ^2^ test were used to investigate differences in distribution of major mechanical complications and predefined variables between male and female operators. Multivariable logistic regression analysis was used to determine association between operator gender and major mechanical complications.

**Results:**

Female operators had a lower rate of major mechanical complications than male operators (0.4% vs 0.8%, *P* = .02), were less experienced (*P* < .001), had more patients with invasive positive pressure ventilation (*P* < .001), more often chose the internal jugular vein (*P* < .001) and more frequently used ultrasound guidance (*P* < .001). Male operators more often chose the subclavian vein (*P* < .001) and inserted more catheters with bore size ≥ 9 Fr (*P* < .001). Multivariable logistic regression analysis showed that male operator gender was associated with major mechanical complication (OR 2.67 [95% CI: 1.26–5.64]) after correction for other relevant independent variables.

**Conclusions:**

The hypothesis was confirmed as differences in distribution of predefined variables between operator genders were found. Despite being less experienced, female operators had a lower rate of major mechanical complications. Furthermore, male operator gender was independently associated with a higher risk of major mechanical complications. Future studies are needed to further investigate differences in risk behaviour between male and female operators.

**Trial registration:**

Clinicaltrials.gov identifier: NCT03782324. Date of registration: 20/12/2018.

## Background

Central venous catheterisation comes with a risk of immediate mechanical complications such as bleeding, cardiac arrhythmia, arterial puncture, pneumothorax and nerve injury [1–4]. Previous studies have reported an incidence of mechanical complications between 1.1% and 18%, where 0.2–2.3% were classified as severe and may have a negative impact on mortality [3, 5–10]. Central venous catheters (CVCs) are both indispensable and common in modern healthcare. Approximately 27 million CVC insertions are performed annually worldwide [11], meaning that mechanical complications contribute significantly to increased morbidity, mortality, and costs.

Real-time ultrasound guidance is strongly recommended for CVC insertions because it both increases success rates and reduces the number of mechanical complications compared with the landmark method [12–23]. However, there are several other factors that also influence the risk of mechanical complications, such as patient characteristics, operator experience, and vascular insertion site [1, 2, 4, 8, 24, 25].

In a recently performed prospective multicentre observational cohort study on incidence and risk factors for mechanical complications after central venous catheterisation (the CVC-MECH trial), the incidence of major mechanical complications was 0.4% in hospitals where real-time ultrasound guidance is the standard of care for central venous access [8]. Interestingly, female operator gender was independently associated with a lower risk of major mechanical complications, which, to the best of the authors’ knowledge, never previously has been reported.

The aim of this study was to further analyse how the predefined major mechanical complications and variables [26] were distributed between male and female operators and to assess if operator gender is associated with major mechanical complications. The hypothesis was that differences in the distribution of the predefined variables between operator genders could be identified.

## Methods

### Setting and participants

All patients ≥ 16 years who received a CVC at any of four emergency care hospitals in Region Skåne, Sweden, from 2 March 2019 to 31 December 2020 were eligible for inclusion. Patients with fictitious social security numbers, arterial catheters accidentally documented as CVC insertions, and CVC insertions with missing insertion dates were excluded. For patients with multiple CVCs during the study period, only one CVC insertion was included, and the inclusion was based on worst case selection. Thus, the CVC insertion with a complication was selected, and if no complication occurred, one of the CVC insertions was randomly chosen. All four hospitals used the same clinical guidelines for CVC insertion [14]. and they all had the same electronic health record system (Melior, Cerner Corporation, North Kansas City, Missouri, USA). One hospital was a university hospital with approximately 1300 beds, and the other three were county hospitals with 200–300 beds each.

### Primary outcome measures

Major mechanical complications defined as 1) bleeding grade 3–4 (bleeding/haemothorax requiring invasive intervention or blood transfusion, and bleeding with life-threatening consequences), 2) persistent nerve injury (clinical signs existing > 72 h), 3) cardiac arrhythmia grade 3–4 (symptomatic arrhythmia requiring urgent medical intervention, and arrhythmia with life-threatening consequences), 4) pneumothorax, and 5) arterial catheterisation [26].

### Predefined variables

The following predefined variables [26] with possible association with mechanical complications were analysed: patient age, gender, body mass index (BMI) and coagulopathy (prothrombin time international normalised ratio > 1.8, activated partial thromboplastin time > 1.3 × normal value (> 43 s), or platelet count < 50 × 10^9^/L), use of invasive positive pressure ventilation, insertion at night (21:00 to 07:00), vascular insertion site (internal jugular vein dexter (dx)/sinister (sin), external jugular vein dx/sin, subclavian vein dx/sin or femoral vein dx/sin), operator gender and experience (number of individual CVC insertions per vascular insertion site prior to study start), ultrasound guidance, catheter bore size (< 9 or ≥ 9 Fr), and number of skin punctures (1 or > 1). All variables were selected based on clinical experience/importance in combination with results from previous studies [3–5, 27].

### Data sources

Previously unpublished data from the CVC-MECH database (Microsoft Excel v. 2013; Microsoft, Redmond, Washington, USA) were used in the present study and all available patients in the database were included. All data were prospectively collected as described in detail in the Methods section of the main study [8, 26]. The manual review of the patient’s electronic health record included text records after the CVC insertion date along with evaluations of postprocedural chest X-rays to identify every mechanical complication that occurred within 24 h after CVC insertion. Furthermore, data regarding previous CVC insertion experience and gender were collected for each operator.

### Statistical methods

Normally distributed data were defined after comparing histograms with the normal distribution curve and median compared with mean and are reported with mean ± standard deviation (SD). Binary and categorical variables are reported as numbers and proportions in percentages. Independent t-test was used when comparing the means of the continuous normally distributed variables ‘patient BMI’ and ‘patient age’. For all other comparisons the χ^2^ test was used. The comparison of the following dichotomised variables between operator genders was based on results from previous studies [1, 3, 4, 8, 27–31]: patient BMI < 20 kg / m^2^, limited operator experience, catheter bore size ≥ 9 Fr., and number of skin punctures (dichotomised to 1 vs > 1). Multivariable logistic regression was used to determine if the association between male operator gender and major mechanical complications remained after adjusting for other relevant independent variables. The number of variables to be included in the multivariable analysis was adapted to the number of major mechanical complications, with a requirement of at least eight events per variable. The inclusion of variables was based on the results from the univariate analyses. As all the defined major mechanical complications only can occur for jugular and subclavian vein catheterisations, femoral vein catheterisations were excluded in the multivariable logistic regression analysis. Results are reported as the odds ratio (OR) with 95% confidence interval (CI). All analyses were performed with SPSS Statistics 28.0.0.0 or 29.0.1.1 (IBM SPSS, Version 28.0 or 29.0. Armonk, NY: IBM Corp). A *P*-value < 0.05 was considered statistically significant.

## Results

**Fig. 1:**
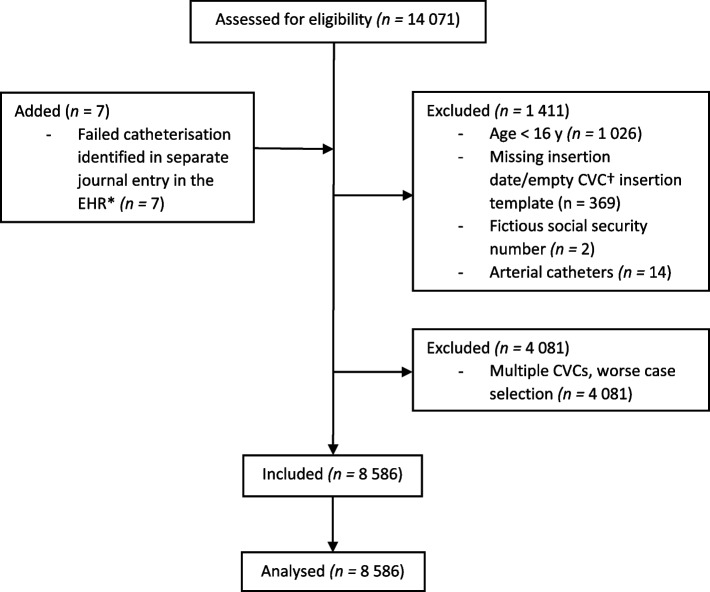
STROBE flow chart

The flow chart in Fig. 1 describes the details of the patient inclusion/exclusion process. A total of 8 586 patients were included. The CVC insertions were performed by 281 individual operators. Patient baseline data, central venous catheter insertion characteristics and operator characteristics are reported in Table 1.

**Table 1:** Patient baseline data, central venous catheter insertion characteristics and operator characteristics

Characteristics	Values,* n* (%) for all variables except Age and BMI, reported with mean (SD)
Patients	8 586 (100)
Female sex	3 535 (41)
Age (yr)	66 (16)
BMI	27 (6.0)
BMI <20	659 (7.7)
Missing	260 (3.0)
Coagulopathy^a^	1 190 (14)
Positive pressure ventilation	4 844 (56)
CVC insertions	8 586 (100)
Insertion technique	
Ultrasound-guided	8 072 (94)
Landmark	230 (2.7)
Change over guidewire	22 (0.3)
Missing	262 (3.1)
Insertion at night^b^	1 232 (14)
Catheter bore size ≥9 Fr	1 387 (16)
Missing	648 (7.5)
Internal jugular vein	7 079 (82)
Subclavian vein^c^	1 134 (13)
Femoral vein	112 (1.3)
External jugular vein	64 (0.7)
Missing	197 (2.3)
>1 skin puncture	1 268 (15)
Missing	301 (3.5)
Individual operators	281 (100)
Female	108 (38)
Operator gender per CVC insertion	
Female	3 053 (36)
Male	5149 (60)
Missing	384 (4.5)
Operator experience per CVC insertion	
Internal jugular vein	7 079 (100)
<100	1150 (16)
≥100	5698 (80)
Missing	231 (3.3)
Subclavian vein	1 134 (100)
<100	503 (44)
≥100	616 (54)
Missing	15 (1.3)
Femoral vein	112 (100)
<100	86 (77)
≥100	19 (17)
Missing	7 (6)

^a^Prothrombin time international normalised ratio > 1.8, activated partial thromboplastin time > 1.3 × normal value (> 43 s), or platelet count < 50 × 10^9^/L

^b^Catheter insertion between 21:00 and 07:00

^c^Subclavian/axillary vein

The distribution of major mechanical complications with regards to operator gender is reported in Table 2. In summary*,* the incidence (95% CI) of major mechanical complications was 0.8% (0.6 – 1.1) for male operators and 0.4% (0.2 – 0.6) for female operators (*P* = .02). The distribution of predefined variables between operator genders is reported in Table 3*.* In summary, female operators were less experienced (*P* < .001), more often used ultrasound guidance (*P* < .001) and had more patients with invasive positive pressure ventilation (*P* < .001) compared to male operators. The choice of insertion site also differed, with more female operators choosing the internal jugular vein (*P* < .001) and more male operators choosing the subclavian vein (*P* < .001). Insertion of large-bore catheters was more common among male operators (*P* < .001).

**Table 2:** Distribution of major mechanical complications between male and female operators

**Major mechanical complications**	Male operator, *n* = 5 149^a^	Female operator, *n* = 3 053^a^	*P*-value
Bleeding grade 3–4^b^, *n* (%)	7 (0.14)	2 (0.07)	.56
Cardiac arrhythmia grade 3–4^c^, *n* (%)	8 (0.16)	1 (0.03)	.20
Arterial catheterisation, *n* (%)	10 (0.19)	5 (0.16)	.96
Pneumothorax, *n* (%)	15 (0.29)	2 (0.07)	.06
Persistent nerve injury^d^, *n* (%)	0 (0)	1 (0.03)	.79
Total, *n* (%)	40 (0.78)	11 (0.36)	**.02**

^a^Missing data for operator gender, *n* = 384 (4.5%). No insertion with a major mechanical complication had missing data on operator gender

^b^Bleeding/haemothorax requiring invasive intervention or blood transfusion and bleeding with life-threatening consequences

^c^Symptomatic arrhythmia requiring urgent medical intervention and arrhythmia with life-threatening consequences

^d^Nerve injury with clinical signs persisting > 72 h

**Table 3:** Distribution of predefined variables between male and female operators

**Predefined variables**	Male operator, *n* = 5 149^a^	Female operator, *n* = 3 053^a^	*P*-value
Patient BMI, mean (SD)	27 (6.0)	27 (5.8)	.46
BMI < 20, *n* (%)	399 (7.8)	227 (7.5)	.61
Patient age, mean (SD)	66 (15)	66 (16)	.06
Coagulopathy^b^, *n* (%)	727 (14)	418 (14)	.59
Positive pressure ventilation, *n* (%)	2931 (57)	1861 (61)	** < .001**
Limited operator experience^c^, *n* (%)	866 (17)	873 (29)	** < .001**
> 1 skin puncture, *n* (%)	753 (15)	479 (16)	.19
Ultrasound-guided, *n* (%)	4909 (95)	2999 (98)	** < .001**
Insertion at night^d^, *n* (%)	637 (12)	398 (13)	.38
Catheter bore size ≥ 9 Fr, *n* (%)	914 (18)	439 (14)	** < .001**
Internal jugular vein, *n* (%)	4158 (81)	2743 (90)	** < .001**
Subclavian vein, *n* (%)	864 (17)	258 (8.5)	** < .001**
Femoral vein, *n* (%)	68 (1.3)	37 (1.2)	.67

^a^Missing data for operator gender, *n* = 384 (4.5%)

^b^Prothrombin time international normalised ratio > 1.8, activated partial thromboplastin time > 1.3 × normal value (> 43 s), or platelet count < 50 × 10^9^/L

^c^< 100 or ≥ 100 individual central line insertions in the chosen vein

^d^Catheter insertion between 21:00 and 07:00

The result from the multivariable logistic regression analysis for major mechanical complication is reported in Table 4. As only a small number of CVC insertions were associated with a major mechanical complication, all predefined variables could not be corrected for. Only the variables that were distributed differently between male and female operators were included, except catheter bore size, which was excluded due to the highest occurrence of missing data. The results showed that male operator gender (OR 2.67 [95% CI: 1.26–5.64]; *P* = .01) and limited operator experience (OR 2.72 [95% CI: 1.44–5.14]; *P* = .002) were independently associated with a higher risk of major mechanical complication.

**Table 4:** Multivariable logistic regression analysis for major mechanical complication

Major mechanical complication (*n* = 45)	Odds ratio	95% CI	*P*-value
Male operator gender	2.67	1.26–5.64	**.01**
Subclavian vein catheterisation	1.37	0.67–2.81	.39
Limited operator experience (< 100)	2.72	1.44–5.14	**.002**
Ultrasound guidance	0.88	0.20–3.79	.86
Positive pressure ventilation	0.64	0.35–1.17	.15
Observations 8 097			

## Discussion

This subgroup analysis of a prospective multicentre observational cohort study, including 8 586 patients, investigated operator gender aspects of major mechanical complications after central line insertions and showed that female operators, despite being less experienced, had a lower rate of major mechanical complications compared to male operators. In addition, multivariable logistic regression analysis for major mechanical complication showed that male operator gender was independently associated with a higher risk of major mechanical complication.

Previous data indicate that operator training and experience are critical for successful cannulation and low complication rates [2, 25, 29, 32–34]. This was confirmed in the previous study on the same cohort where limited operator experience was independently associated with a higher risk of both minor and major mechanical complications [8]. Interestingly, the present subgroup analysis shows that female operators not only had a lower incidence of major mechanical complications than male operators but also were less experienced, a finding that calls for further investigation of explanatory factors.

Differences in patient outcome in relation to caring physician gender have been described in other areas of the medical field. In a recent study by Blohm et al*.* female surgeons were found to have more favourable outcomes than male surgeons in cholecystectomies [35] which is in line with the results from a study by Wallis et al. showing that patients treated by female surgeons had a lower 30-day mortality than patients treated by male surgeons [36]. Similar findings have also been described in the field of internal medicine, were a study by Tsugawa et al. showed lower mortality and readmission rates for patients treated by female internists compared with those treated by male internists [37].

Gender differences in risk behaviour and in willingness to call for help may partly explain these findings [38, 39]. Another possible factor to consider is adherence to clinical guidelines, which Baumhäkel et al*.* have shown to differ depending on physicians’ and patients’ gender [40]. The reason behind these differences needs further investigation.

Ultrasound guidance for central venous access reduces the number of mechanical complications [12–19, 22, 23, 41, 42]. This study showed that female operators used ultrasound guidance to a higher degree than male operators, which could be seen as an explanation for their lower rate of major mechanical complications. However, adjusting for ultrasound guidance in the multivariable logistic regression analysis did not affect the association between male operator gender and higher risk of major mechanical complication.

Female operators had more patients with invasive positive pressure ventilation compared to male operators, but this did not seem to affect the complication rates in this study. In contrast, a retrospective study by Heidemann et al*.* on mechanical complications following CVC insertion, revealed that complications were more common in patients with positive pressure ventilation [27]. However, in a recent randomised clinical trial by Czarnic et al*.* comparing ultrasound-guided catheterisation of the axillary vein with the internal jugular vein in mechanically ventilated patients, no correlation was found between positive end-expiratory pressure or peak inspiratory pressure and success rate. Additionally, the incidence of mechanical complications was low [43].

Differences were also found in the operators’ choice of vascular insertion site. Female operators chose the internal jugular vein to a larger extent, whereas male operators chose the subclavian vein to a larger extent. Several previous studies have shown that catheterisation of the subclavian vein is associated with a higher risk of pneumothorax [2, 3, 7, 8, 26, 32], which could explain the higher rate of major mechanical complications for male operators. However, adjusting for subclavian vein catheterisation in the multivariable logistic regression analysis did not affect the association between male operator gender and higher risk of major mechanical complication.

Previous studies have shown an association between larger bore size catheters and mechanical complications, although mostly in the form of minor bleedings [1, 8, 30]. In this study, male operators were observed to more frequently insert catheters with a bore size ≥ 9 Fr. However, this difference is unlikely to account for their higher rate of major mechanical complications as there was no association between insertion of large bore size catheters and major mechanical complications in the main study on the same cohort [8].

### Limitations

This study has several limitations. First, the study is observational, and the difference in the incidence of major mechanical complications between male and female operators should be confirmed in future studies. Second, the study relies on a strong tradition at the participating hospitals to document every CVC insertion in the medical records, yet some CVC insertions with associated major mechanical complications may not have been recorded. Third, although the predefined variables in the study protocol [26] as well as in the multivariate analysis were selected very carefully, unmeasured confounders may remain. Fourth, considering that the results are hypothesis generating, no correction for multiple testing was undertaken and the results should therefore be interpreted with caution. This concern is partly addressed by the multivariable logistic regression analysis. However, it should be observed that the logistic regression model is susceptible to overfitting due to the limited number of outcomes, further underscoring the need for continued cautious interpretation of the results. Finally, the overall use of ultrasound guidance in this study was very high (95% and 98% for male and female operators, respectively), which makes the results difficult to apply in hospitals where ultrasound guidance is scarce.

## Conclusions

This observational study showed that female operators, despite being less experienced, had a lower incidence of major mechanical complications after CVC insertion than male operators. As proposed in the hypothesis, differences in the distribution of predefined variables between operator genders were identified. However, these differences did not seem to explain the differences in complications rates between male and female operators. When adjusting for the majority of the variables that were distributed differently between the genders in a multivariable analysis, the significant association between male operator gender and a higher risk of major mechanical complication remained. Future studies are needed to confirm or reject this, as well as to gain better understanding of any underlying causes.

## Data Availability

The datasets used and/or analysed during the current study are available from the corresponding author upon reasonable request.

## Abbreviations

CVC: Central Venous Catheter
BMI: Body Mass Index
SD: Standard Deviation
EHR: Electronic Health Record
Dx: Dexter (right)
Sin: Sinister (left)
